# A one stop shop for cost-effectiveness evidence? Recommendations for improving Disease Control Priorities

**DOI:** 10.1186/s12962-019-0175-6

**Published:** 2019-03-20

**Authors:** Matthias Arnold, Susan Griffin, Jessica Ochalek, Paul Revill, Simon Walker

**Affiliations:** Centre for Health Economics, University of York, York, UK

## Abstract

Setting out a health benefits package (HBP) of interventions to be prioritised for funding is an important step towards achieving universal health coverage in low and middle income countries. The 3rd version of the Disease Control Priorities (DCP3) database, and other similar databases, aim to establishing a single point of reference (“one stop shop”) for cost effectiveness evidence to inform HBP design and other policy making. We reflect upon our experiences in using DCP3 for HBP design and offer suggestions for improving the future reporting of cost-effectiveness evidence. We appraise DCP3 based on generalisability, level of detail, and accessibility. We find that DCP and similar initiatives should be commended for the systematic assessment of a vast array of cost-effectiveness studies—the magnitude of such an endeavour is impressive in its own right. However, there are flaws. In future, providing disaggregated estimates of costs and effects, quantifying uncertainty, and systematically assessing the context in which estimates apply would make this evidence more useful for decision makers.

Setting out a health benefits package (HBP) of interventions to be prioritised for funding is an important step towards achieving universal health coverage. Low and middle-income countries (LMIC) experience severe constraints on funding, posing serious challenges for resource allocation. There is growing awareness that HBPs are an important tool for guiding policies aimed towards the goal of universal access. HBP design was noted as a priority topic in the Health Ministers’ Conference of the East, Central, and Southern Africa Health Community [1]. There are recent examples of HBPs being designed and implemented in African [2] and Latin American [3] countries.

Three notable databases aim at providing single points of reference (“one stop shops”) for cost-effectiveness evidence to inform HBP design: the World Health Organization Cost-Effectiveness program (WHO-CHOICE), the Global Health Cost-Effectiveness Analysis Registry by the Tufts Medical Center, and the Disease Control Priorities (DCP) project with its newest version DCP3. DCP3 has received particular attention due to major investment by the Bill & Melinda Gates Foundation and a publication series in the Lancet [4]. Here we reflect upon our experiences in using DCP evidence for HBP design. We highlight problems that analysts may encounter in using DCP3 as a single point of reference from which to extract cost-effectiveness evidence. We offer suggestions for improved reporting of intervention cost-effectiveness studies in future iterations of DCP and other similar initiatives.

## Costs and effects, more than just ratios

The results of published cost-effectiveness studies in DCP3 are typically summarised as the ratio of the incremental cost and benefit between an intervention and a relevant comparator (i.e., incremental cost-effectiveness ratio, ICER). More often than not, the comparator against which the ICER is calculated is not reported in DCP3. This makes it challenging to use this data and assess what estimates really mean in any particular context in which they are applied. In judging the gain from including an intervention in a HBP it is the increment compared to the *appropriate* comparator that is of interest. Appropriate comparators, such as the available relevant alternative interventions and the standard of care, may differ between countries. Therefore, we recommend reporting the comparator when ICERs or incremental costs and benefits are reported.

Ratios can be compared against a decision threshold or estimate of the marginal productivity of healthcare resources to inform decisions about cost-effectiveness. They do not tell us anything about total health benefit or total costs associated with including an intervention in a HBP. In DCP3 effort was made to fill this gap by providing intervention costs in appendices, but these are available for only a fraction of interventions and are unfortunately not disaggregated in full by country or setting. Quantifying the scale of the health benefits and cost of each intervention requires information about per patient cost and benefits alongside local data on the size of the patient population that stands to receive the intervention [5]. We recommend as a minimum the reporting of per patient incremental costs and benefits in these databases. For some situations, the marginal costs and benefits depend on the scale of delivery. In which case, additional information on the scale of implementation and size of the eligible patient population in the underlying studies would be useful in determining their relevance.

## Comparing apples with apples, and discarding rotten apples

When using the results of previously published studies, analysts make judgements about their validity, generalisability and uncertainty. This is crucial to determine the potential for bias and in understanding the confidence that can be placed in results and when extrapolating estimates from one setting to another. In designing a HBP, estimates are required across a wide range of interventions, and it is important to know how far the evidence for each is comparable. These issues are particularly relevant in drawing results from DCP3, which represents a compendium of global evidence.

DCP3 addresses the issue of validity by utilising a checklist for appraising study quality [6], which combines criteria for best practices in study design, methodology and reporting standards. Quality is presented as a sum score. While this allows for the identification of studies with high scores, there is discussion in the literature about the appropriate of using checklist scores as a measure of quality and further it does not elaborate on the nature of any differences between studies [7].

Beyond the scientific rigour addressed in the checklist, methodological choices also influence cost effectiveness results. For example, different choices relating to outcome measures, the time horizon, discounting and perspective can have substantial impact on the cost and effect estimates. Regarding outcomes, the two metrics used most often to inform resource allocation decisions across different diseases are the quality adjusted life year (QALY) and disability-adjusted life year (DALY). Both attempt to do similar things—they both are composite measures reflecting mortality and morbidity. However, the differences between them have been debated and database users may not want to treat them as exchangeable. DCP3 report cost per DALY averted or QALY gained depending upon the outcome measure used in the underlying study, which is DALYs in most instances. DCP can only report what is available, but it should be explicit about which outcomes are included for each study.

Different cost estimates for the same intervention can also be produced by, for instance, using the top-down (splitting up aggregate provider cost or insurance claims into distinct intervention shares) or bottom-up approaches (micro-costing all ingredients necessary). DCP3 addresses this by using the latter if available, but considerable variation nevertheless remains. Again, being explicit about the method by which the costs were produced allows users to consider themselves the extent to which studies are valid and comparable. Factors to report include the time horizon; the discount rates used for costs and benefits and the study perspective.

DCP3 report the country or region of origin of ratio estimates. To be useful across a wide range of countries, DCP3 relies heavily upon assumptions of generalizability. Determining whether cost-effectiveness evidence is generalizable requires assessment of (i) the similarity of the contexts and (ii) the sensitivity of the cost-effectiveness results to aspects that are dissimilar [8]. It is widely recognized that cost-effectiveness results can substantially differ across countries due to variation in (i) epidemiology (e.g. disease prevalence and incidence, conditional survival); (ii) clinical effectiveness; and (iii) economic variables, with both resource use and unit costs depending upon local factors. Reporting at a minimum per patient costs and health effects would allow potential users to assess whether such estimates conform to expectations given local epidemiological, administrative or expenditure data. Reporting further details about the underlying study settings, such as the epidemiologic context, would better enable users to make assessments about generalizability. Even more valuable would be the reporting of sensitivity analysis in the original studies that indicate how the costs and benefits differ with changes in particular parameters.

DCP3 does not systematically address uncertainty, but potential users can refer to the original studies in the appendices to make this assessment for themselves. Even if there is confidence in the validity (i.e. absence of bias) of results, there is inevitable *uncertainty* around any estimates of cost-effectiveness. Uncertainty means that the results of subsequent analyses, for example the recommended HBP, may not provide best value. The true costs and health benefits can differ from the expected results. The likelihood of this informs the level of risk that service delivery cannot be maintained if the true costs exceed available resources, or that significant health benefits are forgone [9]. Without information on the uncertainty around estimates, it is not possible to assess the level of risk, to plan for the possibility of the budget being exceeded nor direct efforts to reducing the key sources of uncertainty. Presentation of point estimates only in the main text of DCP3 risks that users treat all results as if they are subject to the same degree of uncertainty. We recommend that DCP3 make clear where further information on decision uncertainty is available in underlying studies (Boxes 1, 2).

Box 1: Issues of generalisability—the example of emergency caesarean This example illustrates generalisability issues by contrasting the information in the HBP in Malawi on the cost-effectiveness of emergency caesarean care, which is based on a study from a neighbouring country, with the information in DCP3 from a larger geographical context, to illustrate how such estimates might be used in HBP design.

In Malawi, the cost effectiveness of caesarean section for obstructed labour is based on a study in Zambia [10], which estimates that 27.19 DALY are averted at a cost of $300 in 2016 US$ per patient, resulting in an ICER of $11 per DALY averted. In DCP3, ICER estimates range from $1600 to $2600 in 2012 US$ per DALY averted for all LMIC [11]. Incremental averted DALYs and costs are not presented, and identifying the source of this discrepancy requires reviewing the original study used in DCP3. The original study is an international modelling study over 49 countries reporting ICER ranges from $251 to $3462 with 45 of the 49 reporting ICERs below $1000 [12]. The modelling study estimates cost in Malawi to be at $133 and avert 0.48 DALY per caesarean, resulting in an ICER of $274 per DALY averted. Since these numbers are very different from the estimates from the study in Zambia, the methods and assumptions in both studies need to be compared. The main difference lies in the calculation of DALYs, which integrates averted disability and life lost in mothers and neonates in the Zambian study, but reflects only the mother and not the neonate in the modelling study. The Zambian study assumes that neonatal disability weights, the proportion of quality of life lost due to disability, are at 1.00 implying neonatal death. With caesarean section, the mortality rate can be reduced to 11%.

This example demonstrates how important it is to check the appropriateness of a study when using it within a HBP design. Changes in maternal and neonatal mortality from obstructed labour and caesarean sections have major implications for the calculation of DALYs averted. Without knowing the context from which estimates come and assumptions used in the calculation, transferring cost-effectiveness results risks misrepresenting local circumstances and misinforming decisions.

Box 2: Limits of cost-effectiveness ratios—the example of insecticide treated nets for malaria prevention This example illustrates the importance of reporting separately the cost and health impacts from implementing interventions, and not just the ICERs.

DCP3 recommends supplying (insecticide treated nets) ITNs for malaria prevention (compared to standard of care, which is left unspecified) at an ICER range of $61–94 [11]. Supplying households with ITNs and ensuring that pregnant women and children under-5 all slept under ITNs were found to be cost-effective interventions for the Malawian EHP (with ICERs of $33, $34 and $35 per DALY averted respectively) [13]. To determine how much of the budget would be exhausted by the provision of these interventions, information on per patient cost was required in combination with the data on the estimated populations in need in Malawi. This also enabled the calculation of the population health gains associated with that investment. To provide ITNs to all households requiring them would cost $13.4 million and avert 228,000 DALYs; ensuring pregnant women slept under an ITN would cost $3 million and avert 50,000 net DALYs; and ensuring children under-5 slept under an ITN would cost $1 million and avert 17,000 net DALYs.

However, in practice, as a result of health system constraints—including available clinic capacities, trained health care workers and accessibility to health care—it is estimated feasible attainable coverage levels for supplying households with ITNs is only currently approximately 56%, (while, by comparison, full coverage is considered feasible for ensuring pregnant women and children under-5 sleep under an ITN given current constraints). The result is that 128,000 net DALYs are averted out of the 228,000 that could be gained if this was provided at full coverage.

By using per patient incremental costs and health gains, it can be determined that an addition population *net* health gain can be achieved of 79,000 DALYs averted if the health system constraints can be overcome. This can also be used to inform the maximum that Malawi could spend to scale up the coverage of ITNs for households, $4.8 million, before it would be considered cost-ineffective to do so.

## Accessibility and user-friendliness of DCP3

DCP3 should be an easily accessible resource for a wide range of users. However, the presentation of DCP3 results within chapters organized by disease area and without a combined database covering all interventions limits its user-friendliness. Selecting interventions to consider for inclusion in a HBP is fraught with difficulty and the lists in DCP3 that rank interventions based on the ICER could be misleading for the reasons highlighted in this letter. We outline recommendations for improved reporting of cost-effectiveness studies in Box 3.

Box 3: Summary of recommendations

1. When reporting ICERs for an intervention, always specify the comparator.
2. Report per patient costs and per patient benefits, the country and setting to which they relate, and if possible the scale of delivery to which these relate.
3. Report benefits using generic measures of health, and distinguish between DALYs averted or QALYs gained.
4. Report methodological factors including the follow-up/time horizon; the discount rate(s) used for costs and benefits, the study perspective.
5. Report context factors, including health system characteristics on a consistent basis.
6. Provide information on uncertainty.

## Conclusion

DCP and similar initiatives should be commended for the systematic assessment of a vast array of cost-effectiveness studies—the magnitude of such an endeavour is impressive in its own right. However, DCP3 alone does not provide “A concrete set of priorities for universal health coverage” that can reliably be taken up within any one country’s health care system. Its usefulness as a “one stop shop” to inform HBP design for moving toward universal health coverage would be greatly improved by implementing some of the suggestions we have made here in the next iteration.
